# Tribological characteristics of composite brake pads under variable load and speed

**DOI:** 10.1038/s41598-025-33326-7

**Published:** 2026-01-13

**Authors:** Mahmoud A. Essam, Mohamed M. Faragallah, Noha M. Abdeltawab, M. Ali

**Affiliations:** 1 Mechanical Engineering Department, Higher Technological Institute (HTI), 10th of Ramadan City, 44629 Egypt; 2 Faculty of Engineering, City University of Cairo (CUC), New Heliopolis City, Egypt; 3 Mechatronics Engineering Department, Faculty of Engineering, Innovation University, 10th of Ramadan City, Egypt; 4 Faculty of Engineering and Materials Science, German University in Cairo, Cairo, 12613 Egypt

**Keywords:** Brake pads, Coefficient of friction, Abrasive wear, Sliding velocity, Friction materials., Engineering, Materials science

## Abstract

This study investigates the tribological performance of fiber-reinforced composite brake pad materials fabricated using a compression molding technique. The work focuses on evaluating the influence of applied load (10–30 N) and rotational speed (200–1000 rpm) on the coefficient of friction (COF) and wear rate of the developed samples. Experimental tests were conducted using a pin-on-disc tribometer under controlled laboratory conditions to simulate braking contact. The results revealed that both parameters significantly affect the friction and wear behavior of the composites. At lower speeds (200–400 rpm) and loads (10–20 N), the COF remained relatively stable, ranging from 0.63 to 0.72, with a low wear rate below 0.85 mg/N, due to the formation of a compact tribo-film that protected the surface from severe abrasion. As the load and speed increased to 30 N and 800–1000 rpm, the COF increase to 0.795, and the wear rate increased to 1.065 mg/N, indicating the breakdown of the protective layer and the predominance of abrasive and adhesive wear mechanisms. Microscopic analysis using FESEM and EDS confirmed fiber pull-out, particle fragmentation, and localized matrix softening as the main surface features under severe conditions. These findings demonstrate a direct correlation between frictional stability and wear resistance, highlighting that the balance between operating load and sliding speed plays a crucial role in the durability and performance of composite brake pads.

## Introduction

 The main purpose of Brake pads is to transform kinetic energy into thermal energy via friction, therefore reducing or stopping a vehicle’s speed^1^. This process entails intricate interactions between the brake pad and the disc rotor, which must endure severe circumstances, including high temperatures, substantial pressures, and repetitive mechanical stresses. The tribological properties specifically friction and wear resistance of brake pad materials are crucial for preserving Brake pads efficiency and extending component lifespan^2^.

Asbestos was historically utilized in brake pads because of its superior thermal stability, mechanical strength, and economic efficiency. Nonetheless, owing to its established health risks, including carcinogenic properties, its utilization has been prohibited or limited in numerous nations^3^. This has initiated extensive research into alternative materials, such as non-asbestos organic (NAO) composites, semi-metallic formulations, and ceramic-based pads. These options often include binders, fillers, friction modifiers, and reinforcements to achieve ideal performance characteristics. Phenolic resins serve as high-temperature binders, while copper and aramid fibers enhance mechanical strength and thermal dissipation^3,4^.

The wear characteristics of brake pads are affected by various operational aspects, such as sliding velocity, applied load, temperature, and the properties of the friction surface. Research, including Gawande et al., indicates that the wear rate escalates with increased applied stress, and materials such as CL3003 demonstrate markedly reduced wear compared to conventional asbestos-based pads under the same testing conditions^5^. Additionally, the thermal and mechanical properties of brake pads must be refined to mitigate noise, vibration, and particulate emissions, which are significant issues in urban and high-performance settings^6^.

The ongoing progress in materials science and tribology is steering the creation of brake pads that are efficient, robust, and environmentally friendly. The automotive industry is advancing towards elevated safety and emissions regulations, making the selection and optimization of brake pad materials a prominent focus of research and innovation^6^.

## Literature review

The composition of a standard brake pad has four primary types of components: binders, fillers, reinforcements, and friction modifiers. The binder, often phenolic resin, offers thermal stability and mechanical integrity in high-temperature environments^7^. Fillers such baryte (BaSO₄), synthetic graphite, and alumina are incorporated to enhance volume, reduce costs, and modify certain characteristics such as compressibility or fade resistance. Reinforcements like copper and iron powders augment structural integrity and heat conductivity. Friction modifiers, such as abrasives and lubricants like silicon carbide and zinc, assist in regulating the (COF) while minimizing noise and vibrations^6^.

Recent research highlights the tribological characteristics of alternate brake pad materials, evaluating their performance across different loads, sliding velocities, and thermal conditions. Gawande et al. discovered that non-asbestos materials, specifically CL3003, surpassed asbestos in terms of wear resistance and friction stability. Their investigations demonstrated that weight loss in asbestos pads could attain 8–10%, markedly exceeding that of their non-asbestos equivalents^6^.

Asbestos was historically employed in brake pad manufacturing due to its low cost, high thermal stability, and favorable frictional characteristics, which initially rendered it an economically efficient reinforcement material. However, its application has been largely discontinued owing to the severe health and environmental hazards linked to asbestos fiber exposure, including respiratory diseases and cancer^8^. Although asbestos-based pads were once considered cost-effective in terms of raw material price and durability, the long-term economic efficiency is now regarded as negative when accounting for the substantial costs associated with health risks, environmental remediation, and regulatory enforcement. Consequently, contemporary research has shifted toward the development of asbestos-free composites reinforced with natural or synthetic fibers, aiming to achieve an optimal balance between tribological performance, safety, and sustainability^9^.

Binders and other constituents play crucial roles in determining the structural integrity, frictional stability, and overall performance of brake pad composites. The binder, typically a thermosetting resin such as phenolic resin, acts as the primary matrix that holds all ingredients together, ensuring adequate cohesion between reinforcement fibers, fillers, and friction modifiers. It governs the composite’s mechanical strength, thermal resistance, and wear behavior under braking conditions. An optimal binder content ensures strong interfacial bonding and effective load transfer, while excessive or insufficient binder may lead to matrix softening, cracking, or poor adhesion during service^10^.

Other constituents such as reinforcing fibers (e.g., aramid, steel, or natural fibers) enhance the composite’s strength, stiffness, and ability to resist thermal degradation. Friction modifiers (e.g., graphite, metal oxides, or abrasives) are incorporated to regulate the coefficient of friction, improve thermal stability, and prevent fading during high-temperature braking. Fillers like barite or vermiculite contribute to dimensional stability and adjust density and cost, while lubricants such as graphite reduce friction-induced noise and improve wear smoothness. The synergistic interaction among these components particularly the balance between binder rigidity and modifier functionality determines the tribological efficiency, noise–vibration–harshness (NVH) characteristics, and durability of the brake pad^10^.

Kshirsagar and Khairnar investigated both analytical and experimental assessments of the coefficient of friction in brake disc-pad systems^3^. Employing the Greenwood-Williamson contact mechanics model and pin-on-disc testing, they documented a coefficient of friction range of 0.2–0.4, contingent upon load and velocity. Their findings corroborated the model and indicated that material surface roughness and interface pressure significantly affect Brake pads efficiency^11^.

Moreover, recent research has investigated bio-based fillers such as banana peels and carbonized organics as viable eco-friendly alternatives, demonstrating favorable outcomes regarding wear rate and environmental effects^12^. Researchers also investigate aramid fibers, steel wool, and synthetic rubbers to augment mechanical strength while minimizing noise and particulate emissions^13^. Metallic ingredients are commonly used in friction materials for automotive Brake pads systems to increase thermal diffusivity, wear resistance and strength^14^.

The (COF) between the brake pad and rotor surface is significantly affected by the applied load. As loads increase, the actual contact area between surfaces expands due to the deformation of asperities, leading to more equal contact and occasionally a reduced (COF)^15^. Sawczuk et al. noted that an increase in pad clamping force resulted in heightened frictional heating, causing softening at the contact surface and a decrease in the (COF)^16^. For example, at a clamping force of 13 kN, the (COF) was around 0.45; however, it decreased to about 0.38 at 19 kN due to thermally produced lubricious layers and diminished mechanical interlocking at elevated loads^17^.

Panchenko et al. additionally showed that excessive loading diminished the (COF) and led to uneven pad degradation, hence undermining contact uniformity and friction stability over time^18^. The unequal pressure distribution during load application resulted in stress concentrations, facilitating the premature emergence of thermal cracks and localized delamination^19^.

Sliding velocity has a dual impact on the (COF) beginning increments in speed may marginally elevate friction due to enhanced micro-ploughing, whereas extreme velocities generally diminish COF due to thermal effects^20^. Sawczuk et al. reported that the (COF) decreased from roughly 0.46 at 50 km/h to 0.37 at 200 km/h in a railway disc brake test apparatus^16^. This decline is ascribed to the thermal glazing of the pad surface and the loss of mechanical adhesion at elevated velocities^16^.

Abrasive wear rate generally increases with applied load. In the same pin-on-disc tests, the wear volume of the specimen increased from 0.85 mm³ to 1.42 mm³ as the load increase from 30 N to 70 N, indicating higher energy dissipation and micro-cutting mechanisms at elevated loads^21^. The wear rate (calculated as volume loss per sliding distance) doubled under this load increment, signifying load sensitivity in abrasive conditions.

This inverse relationship was further corroborated by a mid-band frequency vibration analysis, in which elevated sliding speed diminished signal amplitude due to decreased interfacial friction^22^. At elevated velocities, stick-slip transitions diminished, leading to a more stable yet lowered coefficient of friction profile^22^.

The sliding velocity significantly influences the degree of abrasive wear, however the correlation may be non-linear^23^. As velocity escalates, surface temperature increases, affecting oxidation and microstructural alterations in the pad material. Sawczuk et al. (2022) indicated that wear escalated with velocity up to 120 km/h, reaching a maximum mass loss of 1.08 g, subsequently decreasing to 0.98 g at 160 km/h, implying the development of protective oxide layers at elevated temperatures^16^.

Rotational speed (RPM), as a proxy for sliding velocity in rotating systems, also impacts the COF. As RPM increases, the surface temperature rises, which often leads to thermal degradation of the contact interface. In a disk brake test, increasing RPM from 300 to 800 led to a decrease in COF from 0.43 to 0.36 due to thermal softening and possible formation of third-body layers^24^. However, the effect plateaued beyond 800 RPM, suggesting a dynamic balance between heating and material adaptation^25^.

This study aims to evaluate the effects of applied load, sliding velocity, and rotational speed on the coefficient of friction and wear rate of a semi-metallic brake pad composite, to optimize Brake pads performance and material durability under varying operating conditions.

## Experimental section

### Brake pads friction materials

This study involved the fabrication of non-asbestos organic (NAO) brake pad compositions, consisting of thirteen constituents as detailed in Table 1, utilizing a powder metallurgy approach. The manufacturing process adhered to a systematic sequence to guarantee optimal material properties and performance. The raw materials were blended in a rotating mixer at 90 rpm for 3 h to ensure uniform distribution of the ingredients. A backing plate was prepared to ensure a stable substrate for the adhesion of composite materials. The mixed powder was subjected to perform compaction to attain initial densification. A hot compaction process was subsequently conducted using a hydraulic press within a die at a pressure of 20 MPa. In the process of hot compaction, the upper and lower molds were held at temperatures of 168 °C and 177 °C, respectively, for a duration of 6 min to facilitate robust interparticle bonding and improve mechanical integrity.

**Fig. 1:**
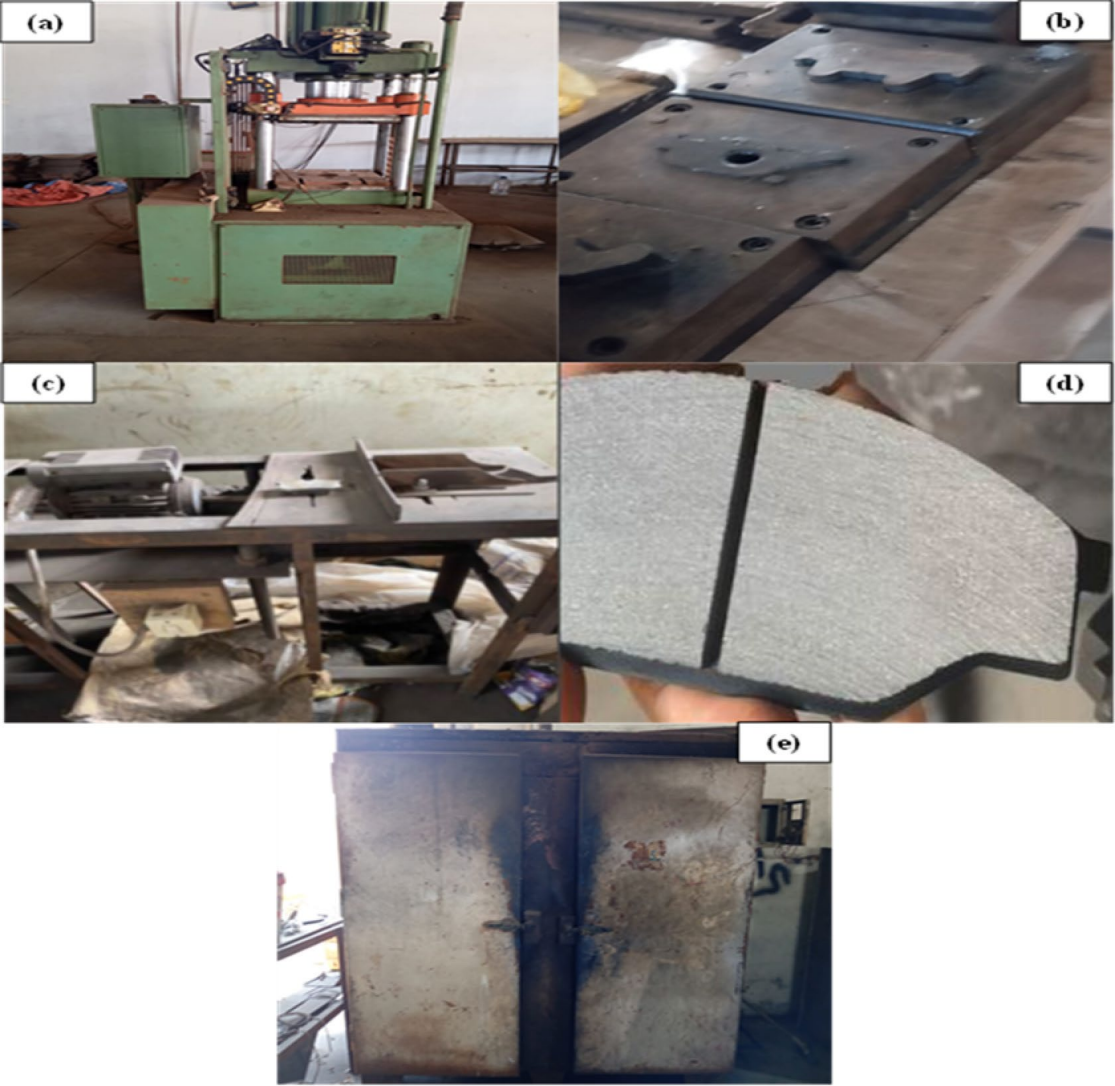
Real images of the brake pad manufacturing process: (**a**) Hydraulic hot press used for compression molding, (**b**) mold assembly for forming brake pads, (**c**) grinding and finishing machine, (**d**) produced brake pad sample after finishing, and (**e**) curing oven used for post-curing treatment.

A post-treatment was conducted at 170 °C for 2 h to enhance the stability of the thermoset matrix, thereby improving its toughness and thermal stability. The fabricated samples underwent a finishing process to enhance their surface and dimensional properties, ensuring adherence to rigorous quality and performance standards. The prototype, referred to as the tested sample, functions as a benchmark for comparative analysis and performance assessment. Figure 1 presents a schematic representation of the manufacturing process.

The reference brake pad used for comparison in this study was an aftermarket semi-metallic pad, which served as a benchmark to evaluate the performance of the developed asbestos-free composite pads. The newly fabricated composites were reinforced with aramid fibers, magnesium oxide (MgO), and zirconium oxide (ZrO₂) to enhance their mechanical and tribological performance. The incorporation of aramid.

fibers improved the structural integrity and resistance of the material, while MgO contributed to thermal stability and reduced matrix degradation at elevated temperatures. In addition, ZrO₂ enhanced wear resistance and helped stabilize the coefficient of friction during braking operation.

**Table 1:** Formulation of chemical composition for the tested sample of friction material.

Element	Graphite	Wire	Rock wool	Rubber	Lime	Barite	Vermiculite	Phenolic Resin	Zirconium oxide	Aramid fiber (3 mm)	SiC	MgO	Coke
Weight%	5.5	5	5	7	5	26.5	6	11	4	7	3	7	8

The manufacturing process of the brake pads involves several sequential stages, as illustrated in Fig. 1 (a – e), using actual experimental setup photographs instead of schematic representations. Initially, the raw materials, including reinforcement fibers, binders, abrasives, lubricants, and fillers, are accurately weighed and mixed thoroughly to achieve a homogeneous and uniform composite blend. This step ensures proper dispersion of all constituents, which is critical for consistent material performance. The prepared mixture is then placed into a pre-heated mold cavity, as shown in Fig. 1(b), and subjected to compression molding using the hydraulic hot press presented in Fig. 1(a). The molding is conducted under controlled temperature and pressure conditions to allow the resin to flow and bond effectively with the fibers, forming the brake pad shape. After demolding, the semi-cured pads are transferred to the curing oven shown in Fig. 1(e) for post-curing at a specified temperature and time to complete the cross-linking process and improve the mechanical integrity and thermal stability of the composite. Following curing, the pads are ground and finished using the machine illustrated in Fig. 1(c) to achieve the desired dimensions, surface smoothness, and uniformity. Grooves and chamfers are added during this stage to enhance frictional stability and heat dissipation. Finally, the completed brake pad, shown in Fig. 1(d), is inspected for any surface or dimensional defects and prepared for subsequent physical and tribological testing to evaluate its performance characteristics such as hardness, wear resistance, and coefficient of friction.

### Microstructure and hardness test

Table 2 presents the detailed composition and technical specifications of the materials used in fabricating the asbestos-free composite brake pads. Each constituent was selected based on its functional role in optimizing the mechanical and tribological performance of the composite. The phenolic resin (Novolac type PF-234) served as the binder matrix, providing structural cohesion and thermal stability. Date palm fibers were introduced as natural reinforcements to enhance strength, wear resistance, and eco-friendliness. Magnesium oxide (MgO) acted as a thermal stabilizer, improving heat resistance and reducing degradation of the resin at elevated temperatures, while zirconium oxide (ZrO₂) functioned as a wear enhancer that stabilized the coefficient of friction during braking. Graphite served as a solid lubricant, promoting steady frictional behavior and reducing surface damage. Barite (BaSO₄) was incorporated as an inert filler to control density and improve dimensional stability, whereas steel wool, when used, provided secondary metallic reinforcement to further increase load-bearing capacity and heat dissipation.

**Table 2:** Composition, function, and key specifications of the materials used in brake pad fabrication.

Material	Type / Function	Grade / Specification	Key Technical Details
Phenolic resin	Binder / Matrix	Novolac type PF-234	Softening point: 98–105 °C; Recommended cure: 170–180 °C; Flow: 22–28 mm
Natural fiber	Reinforcement	Date palm fiber (chopped, 5 mm length)	Cellulose content: ~62%; Density: 1.35 g/cm³; Dried at 80 °C for 2 h
Magnesium oxide (MgO)	Filler / Thermal stabilizer	Industrial grade, ≥ 99% purity	Melting point: 2852 °C; Particle size: ≤75 μm
Zirconium oxide (ZrO₂)	Filler / Wear enhancer	Technical grade	Particle size: ≤50 μm; Purity ≥ 98%; Melting point: 2715 °C
Graphite	Solid lubricant / Friction modifier	Natural flake, 99% purity	Particle size: ≤45 μm; Density: 2.25 g/cm³
Barite (BaSO₄)	Inert filler / Density adjuster	Micronized, industrial grade	Purity: ≥96%; Particle size: ≤75 μm
Steel wool (optional)	Secondary reinforcement	Low-carbon, 0.1 mm diameter	Tensile strength: 480 MPa

### Microstructure and hardness test

Samples with dimensions the same as those prepared for microscopy were utilized to evaluate the hardness properties of the brake pad specimens. The hardness of the developed brake pad specimens was measured in accordance with ASTM D785 (Standard Test Method for Rockwell Hardness of Plastics and Electrical Insulating Materials) using a Rockwell Hardness Tester (Model HR-430). The Rockwell ‘C’ scale (HRC) was selected because the brake pad composites exhibited relatively high stiffness and contained hard ceramic fillers (MgO, ZrO₂, barite), which made softer scales such as HRB unsuitable. The ‘C’ scale provides better resolution and consistency for materials with higher hardness levels typical of semi-metallic and fiber-reinforced friction composites. Four readings were taken per specimen (150 × 100 mm), and the average value was reported to minimize local variation. Microstructural characterization utilized field emission scanning electron microscopy (FESEM; Carl Zeiss Sigma AG, Oberkochen, Germany) and laser scanning confocal microscopy (LSCM; VK-200, Keyence Ltd., Osaka, Japan). These techniques facilitated a comprehensive evaluation of surface morphology, microstructural features and phase morphology, and phase distribution in the brake pad composites. The FESEM analysis yielded high-resolution insights into particle bonding and interfacial integrity, whereas LSCM enabled three-dimensional topographic mapping of the surface. These methodologies clarified essential connections among microstructure, mechanical performance, and compositional homogeneity.

### Wear test

**Fig. 2:**
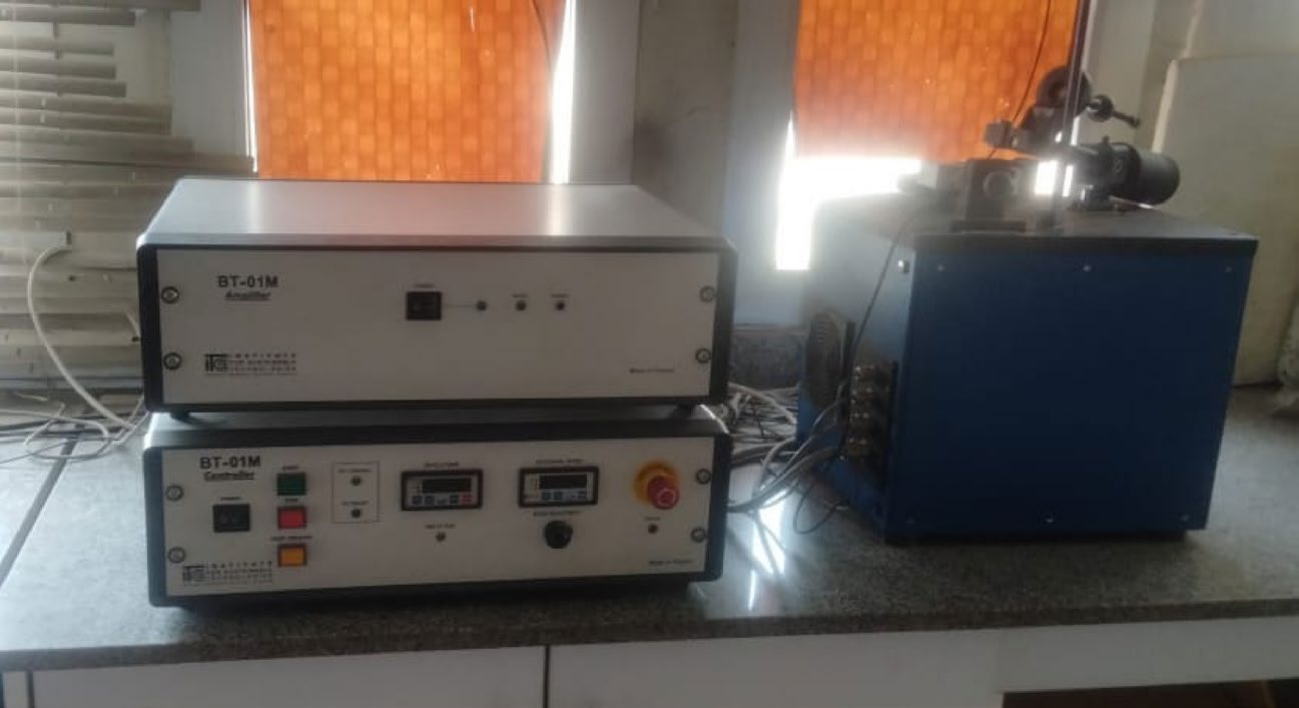
Pin-on-Disc tribometer used for wear and friction analysis.

Measurements of wear and friction coefficients were performed Pin – on – Disc Apparatus. In the testing phase, the brake pad sample was placed in contact with a rotating brake drum, which operated at incremental rotational velocities of 200, 300, 400, 500, 600, 700, 800, and 1000 rpm as shown in Fig. 2. The test duration facilitated a comprehensive assessment of the material’s tribological behavior, including friction and wear characteristics, as well as wear resistance under conditions that simulate real-world automotive applications. In this study, the general testing procedure and evaluation parameters were based on the SAE J661 standard; however, the rotational speed varied between 200 and 1000 rpm to systematically investigate the effect of sliding velocity on the friction and wear behavior of the developed brake pad materials.

A pin-on-disc tribometer was utilized for supplemental analysis to measure wear rate and friction coefficient. Specimen pins with specified dimensions (6 mm in diameter and 18 mm in height) were fabricated and affixed to the machine. The testing parameters, such as applied load, sliding speed, and test duration, were meticulously regulated. The pin was applied to the rotating disc under specified conditions, and the instantaneous friction coefficient was documented at 20-second intervals throughout a 20-minute duration. This method guaranteed high-resolution observation of tribological dynamics during the test. All experimental procedures were conducted at the Mechanical Testing and Materials Characterization Laboratory of the at the Central Metallurgical Research and Development Institute (CMRDI), Egypt.

## Results and discussions

### Microstructure examination

**Fig. 3:**
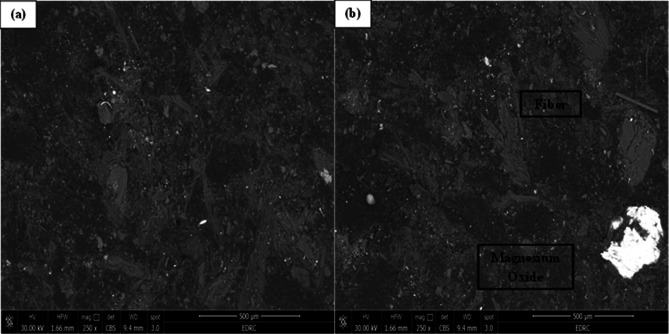
SEM micrographs of the fabricated brake pad composite showing the distribution of reinforcing constituents: (**a**) overall microstructure at 250× magnification and (**b**) localized regions highlighting fiber and magnesium oxide particles within the matrix.

The supplied SEM micrographs revealed inadequate and insufficient bonding between certain constituents, while observable fibers were identified across the fractured surface, indicating weak interfacial adhesion in localized regions. Figure 3a presents an overview of the composite, emphasizing the uneven distribution of elements and the occurrence of microstructural flaws, including micro-cracks and voids. These characteristics indicate inhomogeneity within the matrix, perhaps resulting from inadequate mixing or insufficient adhesion between the reinforcement and the matrix. The SEM micrographs reveal a relatively coarse morphology with visible fiber pull-outs and fragmented particles, suggesting surface degradation due to severe wear; however, quantitative surface roughness was not measured in this study^26,27^. The uneven configurations and dispersed characteristics of the implanted particles further suggest a multiphase material engineered to endure mechanical forces and thermal fluctuations during Brake pads.

Figure 3b offers a detailed view with annotated sections delineating several phases within the composite. A fibrous structure is integrated in the matrix, indicating the reinforcement phase potentially organic or inorganic fibers included to enhance the mechanical strength and toughness of the brake pad. These fibers function to disperse stresses during Brake pads and mitigate crack formation. A bright white inclusion described as magnesium oxide (MgO), a prevalent addition in friction materials, is also noted. Owing to its elevated atomic number, MgO exhibits increased brightness in backscattered electron microscopy and is commonly incorporated to enhance thermal conductivity, wear resistance, and frictional stability^28^. The well-dispersed presence indicates good integration into the matrix; however, perfect homogeneity may not be attained, as certain places exhibit clustering or uneven distribution.

The composite’s microstructure, as depicted in these SEM pictures, demonstrates a purposeful amalgamation of hard particles and fiber reinforcement within a polymer or resin matrix. The observable fissures and delamination in Fig. 3a may signify areas of inadequate fiber-matrix adhesion, potentially undermining performance under severe thermal and mechanical stresses^28^. The SEM micrographs in Figure (3-b) qualitatively illustrate the presence and distribution of magnesium oxide particles and reinforcing fibers within the matrix, indicating a uniform microstructural dispersion achieved through proper mixing and molding. The SEM examination indicates that the brake pad sample has the characteristic structural complexity of a reinforced composite material, with microstructural characteristics that significantly affect its tribological performance and operational longevity^29^.

The coarse surface morphology, along with observable fiber pull-outs and fragmented particles, indicates active wear mechanisms primarily a combination of abrasive and adhesive wear typically observed in high-friction applications such as braking systems. The uneven configuration and dispersed distribution of the embedded particles further indicate a multiphase composite structure specifically engineered to withstand mechanical stresses and thermal fluctuations encountered during braking operations.

The fiber dimension is a critical parameter influencing the mechanical integrity and tribological performance of fiber-reinforced composites. In the present study, both the fiber length and diameter were carefully optimized to achieve effective interfacial bonding and uniform stress distribution within the matrix during frictional loading. Inadequately short or fine fibers tend to weaken load transfer and reduce wear resistance, while excessively long or coarse fibers may lead to agglomeration, fiber pull-out, and microstructural inhomogeneity. Proper optimization of the fiber geometry promotes a well-integrated microstructure, enabling efficient stress transfer and microcrack bridging during sliding. Moreover, suitable fiber dimensions facilitate the stable formation of a protective tribo-film on the contact surface, which enhances the coefficient of friction and wear resistance, consistent with the findings of Singh et al. Therefore, in this study, the fiber dimension was identified and treated as a key design factor governing the overall performance and durability of the developed brake pad composites.

### Hardness measurements

**Fig. 4:**
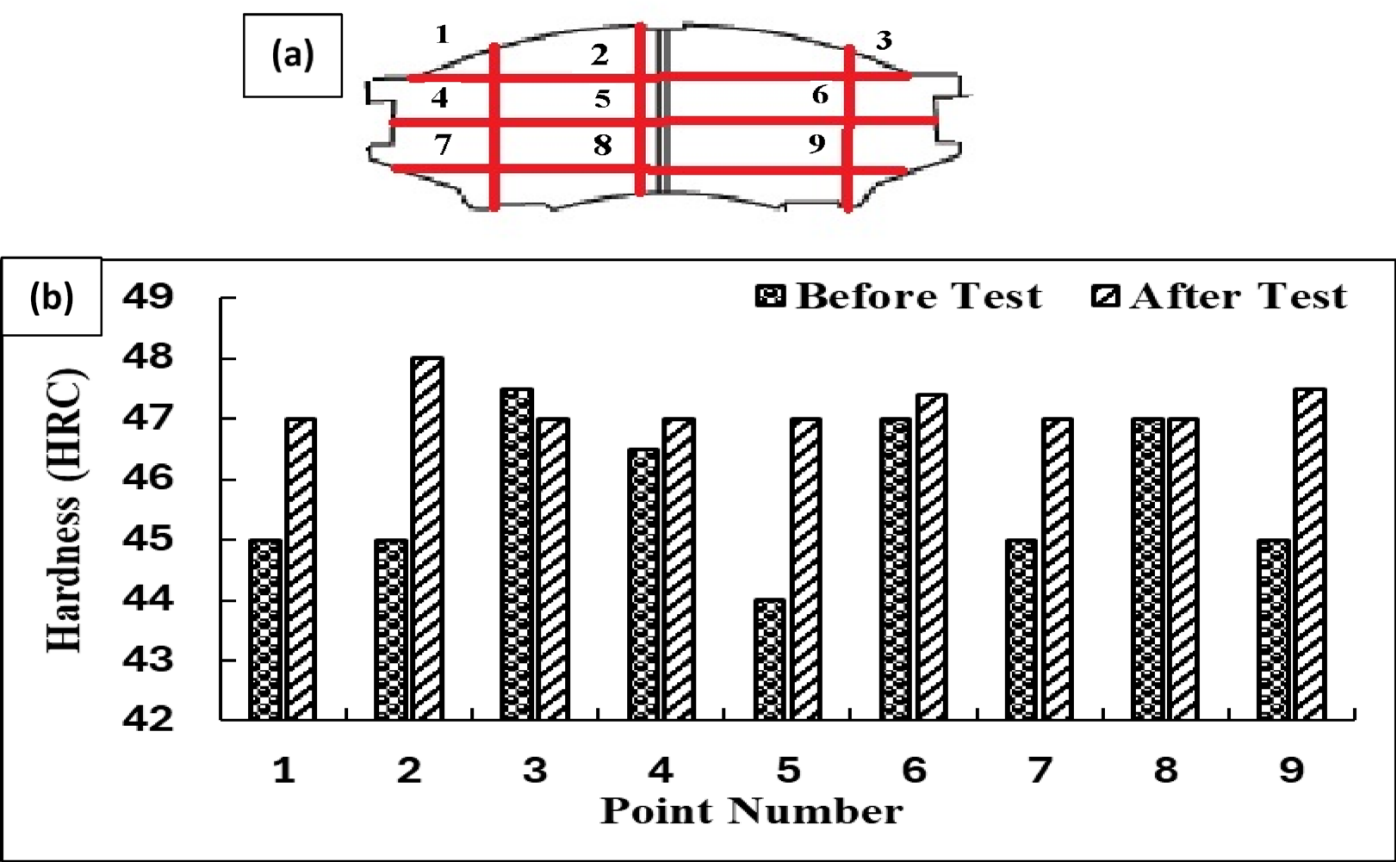
Hardness Distribution Before and After Testing Across Selected Points (**a**) show intersection points of measurements and (**b**) HRC values.

Figure 4 depicts the Rockwell Hardness (HRC) values of a brake pad formulation consisting of silicon carbide (SiC), 3-mm fibers, and magnesium oxide (MgO), assessed prior to and subsequent to tribological testing. The findings demonstrate a uniform rise in hardness throughout all areas of the material post-testing, signifying strain hardening due to frictional heating and mechanical stress encountered during braking. The observed increase in surface hardness post-testing can be attributed not to classical strain hardening of the bulk composite, but rather to the formation of a compacted and work-hardened tribo-layer at the contact interface during sliding. The presence of SiC contributes to this effect by providing high hardness and resistance to plastic deformation, thereby promoting micro-compaction of wear debris and surface densification under repeated frictional stress. Meanwhile, MgO enhances the composite’s thermal stability and grain boundary integrity, limiting thermal softening and microstructural degradation at elevated temperatures. Together, these mechanisms result in a localized increase in surface hardness and improved wear resistance after testing^30^.

Before testing, minor variations in HRC values were observed due to the non-uniform distribution of fibers and fillers. After tribological testing, the hardness increased uniformly across all regions, indicating the material’s improved resistance to wear and sustained mechanical stability under repeated loading^31^.

The post-test hardness improvement is attributed to synergistic effects: silicon carbide increased surface durability through its high hardness, magnesium oxide enhanced thermal stability and limited microstructural degradation, and the 3 mm fibers distributed stresses uniformly, reducing localized wear and crack propagation^32^. The increase in hardness is associated with microstructural densification and the development of a friction-induced protective tribolayer on the contact surface, indicating the composite’s adaptive response to challenging operational conditions. The findings underscore the substantial roles of SiC, MgO, and fiber reinforcement in enhancing the durability and reliability of brake pad materials, rendering this formulation exceptionally appropriate for high-performance braking systems that necessitate prolonged service life and wear resistance^32^.

### Effect of applied load on coefficient of friction

**Fig. 5:**
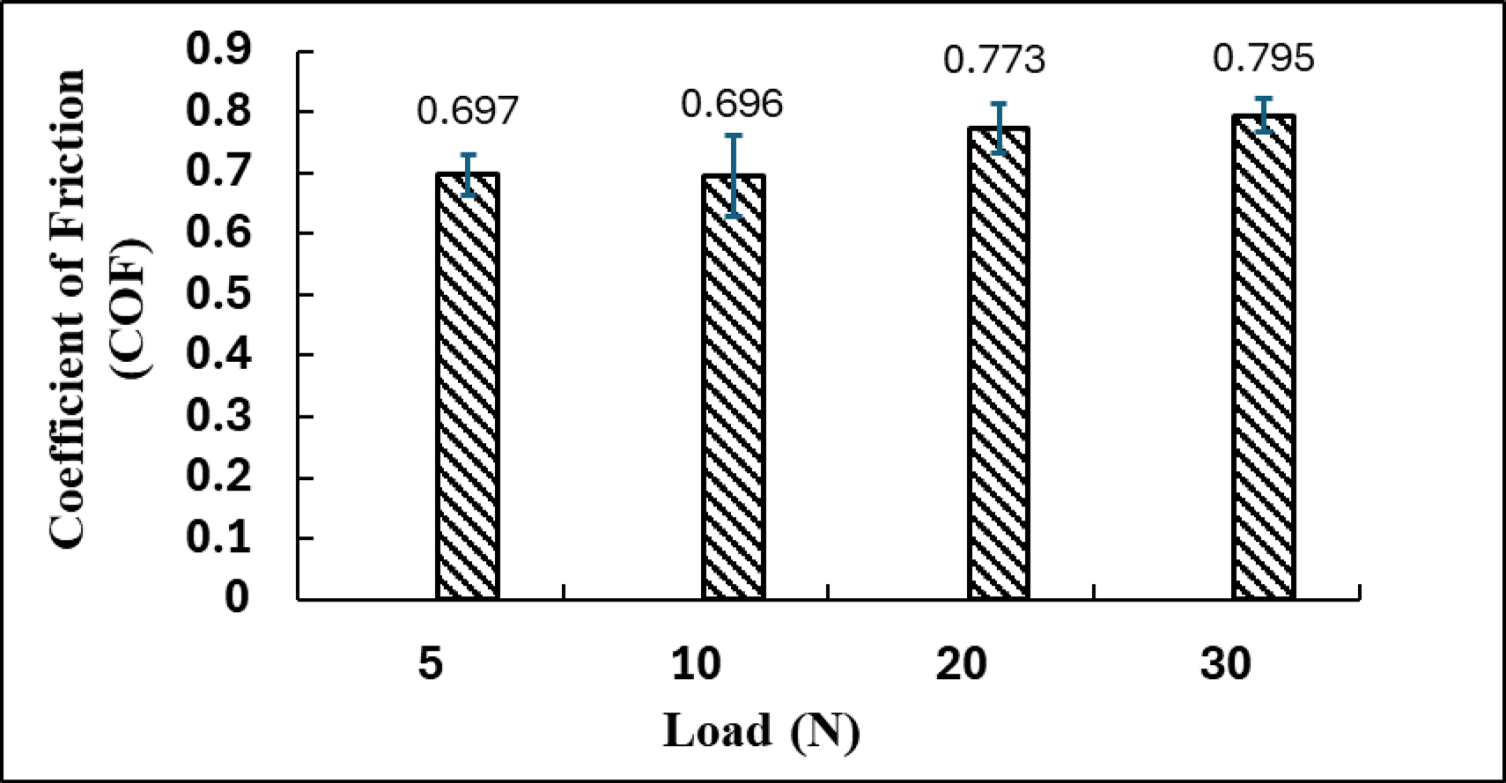
COF Behavior Under Different Normal Loads.

Figure 5 depicts the behavior of the (COF) for a brake pad composite material subjected to different applied normal loads of 5 N, 10 N, 20 N, and 30 N. The (COF) is an essential metric for assessing braking performance, as it indicates the material’s capacity to withstand sliding under pressure; a higher COF typically corresponds to improved braking efficiency. Figure 5 presents the average COF values along with the associated error bars, indicating the standard deviation or variability in the measured values.

At a load of 5 N, the composite demonstrates a coefficient of friction of 0.697, indicating the material’s initial frictional performance under low contact pressure. This value is classified as moderately high, suggesting that the brake pad surface effectively resists sliding. This effectiveness is likely attributable to factors such as surface roughness, particle interactions, and the initial development of a tribolayer^33^. At 10 N, the coefficient of friction decreases marginally to 0.696, indicating negligible change. The observed minor difference indicates stability in contact conditions within this load range, suggesting that the brake pad material can sustain consistent performance under light to moderate loads. The minimal deviation indicates a stable interface, characterized by the absence of excessive wear or smearing at this stage, thereby maintaining the integrity of the contact surface^33,34^.

With an increase in the applied load to 20 N, the (COF) increases markedly to 0.773. The increase indicates a change in the tribological behavior of the material, suggesting that more intense interfacial interactions are occurring. This may result from various phenomena: improved mechanical interlocking of surface asperities, heightened activation of reinforcing particles (e.g., SiC and MgO), and potentially the emergence of mild thermal effects that cause softening or flow of the matrix phase, thereby enhancing adhesion with the counter surface. This behavior indicates that the material transitions to a more effective frictional regime, enhancing its resistance to motion through optimized component engagement^34^.

Under the maximum applied load of 30 N, the (COF) attains a maximum value of 0.795, signifying optimal frictional performance across all evaluated conditions. This trend indicates that the brake pad composite exhibits significant pressure sensitivity and reacts favorably to elevated normal loads, likely attributable to tribo-film formation compaction, tribolayer stabilization, and the mitigation of micro-cracking or delamination under prolonged loading conditions^35^. The development of a stable, protective third-body layer from wear debris may enhance contact adhesion and decrease material loss. This finding is significant for practical braking applications, where materials experience repeated high loads and must reliably provide effective stopping power^36^.

**Fig. 6:**
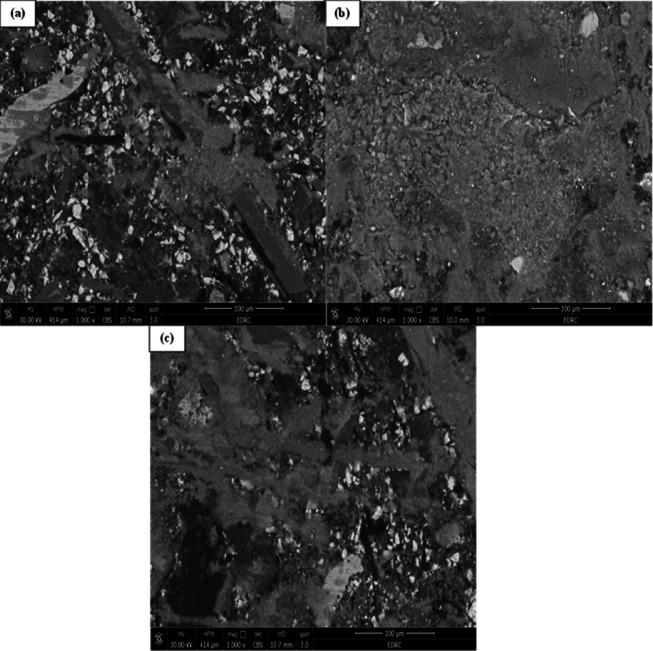
SEM micrographs of the worn surface of the brake pad composite at different rotational speeds: (**a**) 200 RPM – localized abrasive grooves and detached debris; (**b**) 400 RPM – compacted and continuous tribo-film (smooth grey region) formed by smeared wear debris and resin; (**c**) 800 RPM – film breakdown, cracks, and exposed reinforcement particles due to severe thermal stress.

Further examination of the worn surface under a 20 N load and 0.6 m/s sliding condition confirmed the formation of a compacted third-body layer, as shown in the newly added Fig. 6d. The high-magnification SEM micrograph reveals smooth and densified tribo-film bridging surface asperities, formed through the compaction and smearing of fine wear debris generated during sliding. This film acts as a protective interface that temporarily stabilizes the coefficient of friction by maintaining a continuous shear zone between the composite and the counter face. The relatively high COF value (0.795) observed at 30 N is therefore attributed mainly to enhanced asperity interlocking and particle compaction rather than severe counter body abrasive wear. Since the test was conducted against a hardened steel disc (≈ 55 HRC), the likelihood of counter face ploughing was minimized. Nevertheless, in practical braking applications involving cast iron rotors, such frictional conditions may lead to higher disc wear. Although the present work quantified only pad wear due to the limitations of the laboratory-scale setup, future investigations will assess the combined pad–disc wear behavior to provide a comprehensive evaluation of the tribo system.

### Effect of sliding velocity on coefficient of friction

**Fig. 7:**
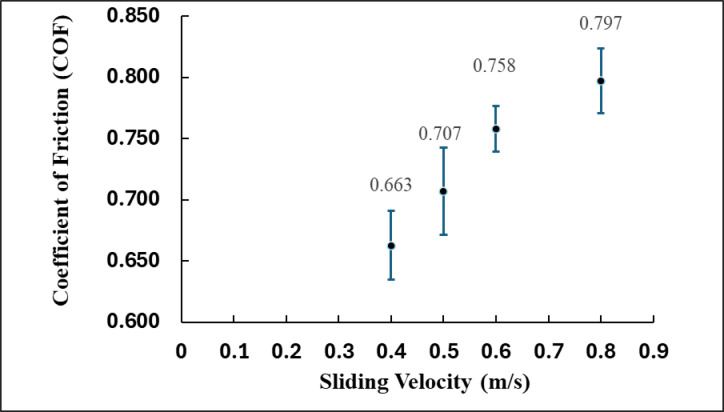
Relationship between sliding velocity and frictional behavior.

Figure 7 demonstrates the variation of the (COF) for a brake pad composite in relation to sliding velocity, which spans from 0.4 m/s to 0.8 m/s. The data points indicate a clear increasing trend in COF with increasing velocity, suggesting enhanced frictional performance at elevated operational speeds. Each data point includes error bars that indicate the variability or uncertainty in the measured values, thereby reflecting the reliability and repeatability of the test^37^.

At a sliding velocity of 0.4 m/s, the (COF) is recorded at 0.663, representing the lowest value observed throughout the entire test range. The observed lower friction level can be ascribed to relatively mild interfacial contact, restricted thermal activation, and a less developed third-body layer formation. Furthermore, at this velocity, the kinetic energy applied is minimal, potentially resulting in inadequate engagement between the composite constituents and the counter surface, thereby diminishing frictional interaction. The error bar at this velocity is one of the largest, suggesting increased variability in frictional behavior, likely attributable to unstable contact or microstructural inconsistencies^38^.

At a velocity of 0.5 m/s, the (COF) increases to 0.707, indicating a rise of approximately 6.6% relative to the value at 0.4 m/s. This increase indicates the emergence of a more stable tribological interaction between the brake pad and the sliding surface. The surface temperature at this velocity is likely adequate to induce mild softening or flow of the matrix, resulting in improved surface conformity and enhanced mechanical interlocking.

At a velocity of 0.6 m/s, the (COF) attains a value of 0.758, representing a 7.2% increase compared to the value of 0.5 m/s. The ongoing upward trend can be linked to a more effective tribo-film formation on the contact surface, as the increase in temperature promotes plastic deformation and compaction of wear debris. This behavior generally leads to enhanced adhesion and shear strength at the interface, thereby increasing friction.

The maximum (COF) is observed at a sliding velocity of 0.8 m/s, attaining a value of 0.797. This indicates a 20.2% increase relative to the initial coefficient of friction at 0.4 m/s. At this velocity, considerable thermal and mechanical stresses are exerted on the material. The brake pad composite exhibits interfacial stability, attributed to the synergistic effects of reinforcing phases including SiC, MgO, and fiber reinforcement. The microstructure of the composite likely enhances thermal conductivity and structural stability, thereby preventing degradation or smearing of the contact layer, which could otherwise result in reduced friction.

The coefficient of friction (COF) increased from 0.663 at 0.4 m/s to 0.797 at 0.8 m/s, representing an overall rise of approximately 20.2%. This increase is primarily attributed to enhanced interfacial shear interaction and localized frictional heating at higher sliding velocities. The elevated temperature promotes the breakdown of weak surface films and exposes fresh, rougher surfaces, resulting in stronger mechanical interlocking between the asperities of the pad and disc. However, as reported by Singh et al.^9^.such an increase in COF at high speeds often indicates a transition from mild to severe wear, caused by unstable tribo-film formation and oxidative degradation. The frictional heat generated may alter the near-surface microstructure, leading to fiber–matrix debonding and fragmentation of wear debris, which subsequently accelerates abrasive and fatigue wear mechanisms. Therefore, while the higher COF reflects stronger interfacial contact, it also suggests that the wear mechanism requires optimization particularly through stabilizing the tribo-film and enhancing heat dissipation. The incorporation of thermally conductive additives or surface-modifying agents, as recommended by Singh et al. could mitigate excessive heat accumulation and maintain a steady-state friction response, ensuring an improved balance between frictional performance and wear resistance^39^.

The tribological results revealed a clear correlation between the coefficient of friction (COF) and the wear rate of the developed brake pad composites under varying loads and rotational speeds. At a load of 10 N and speed of 200 rpm, the COF was approximately 0.63, with a corresponding wear rate of 0.582 mg/N, indicating stable friction and minimal material removal due to the formation of a protective tribo-film. As the load increased to 20 N and the speed to 400 rpm, the COF increase slightly to 0.72, while the wear rate increased to 0.845 mg/N, suggesting partial breakdown of the film and the initiation of abrasive wear. At the highest test condition of 30 N and 800 rpm, the COF further increased to 0.795, accompanied by a wear rate of 1.065 mg/N, confirming that elevated thermal and mechanical stresses led to fiber pull-out, surface fragmentation, and increased material loss.

This trend confirms that higher friction coefficients are directly associated with greater wear rates, primarily due to the combined effects of adhesive and abrasive wear mechanisms. The rise in COF with load indicates enhanced interfacial interaction and mechanical interlocking between the pad and disc surfaces, whereas the parallel increase in wear rate reflects the dominance of micro-cutting and particle detachment at higher stress levels. Therefore, maintaining an optimum COF between 0.6 and 0.75 is critical for ensuring effective braking performance while minimizing surface degradation. The correlation between friction and wear behaviors demonstrates the importance of balancing mechanical hardness, reinforcement dispersion, and thermal stability to achieve durable and consistent brake pad performance.”

### Abrasive wear based on applied load

**Fig. 8:**
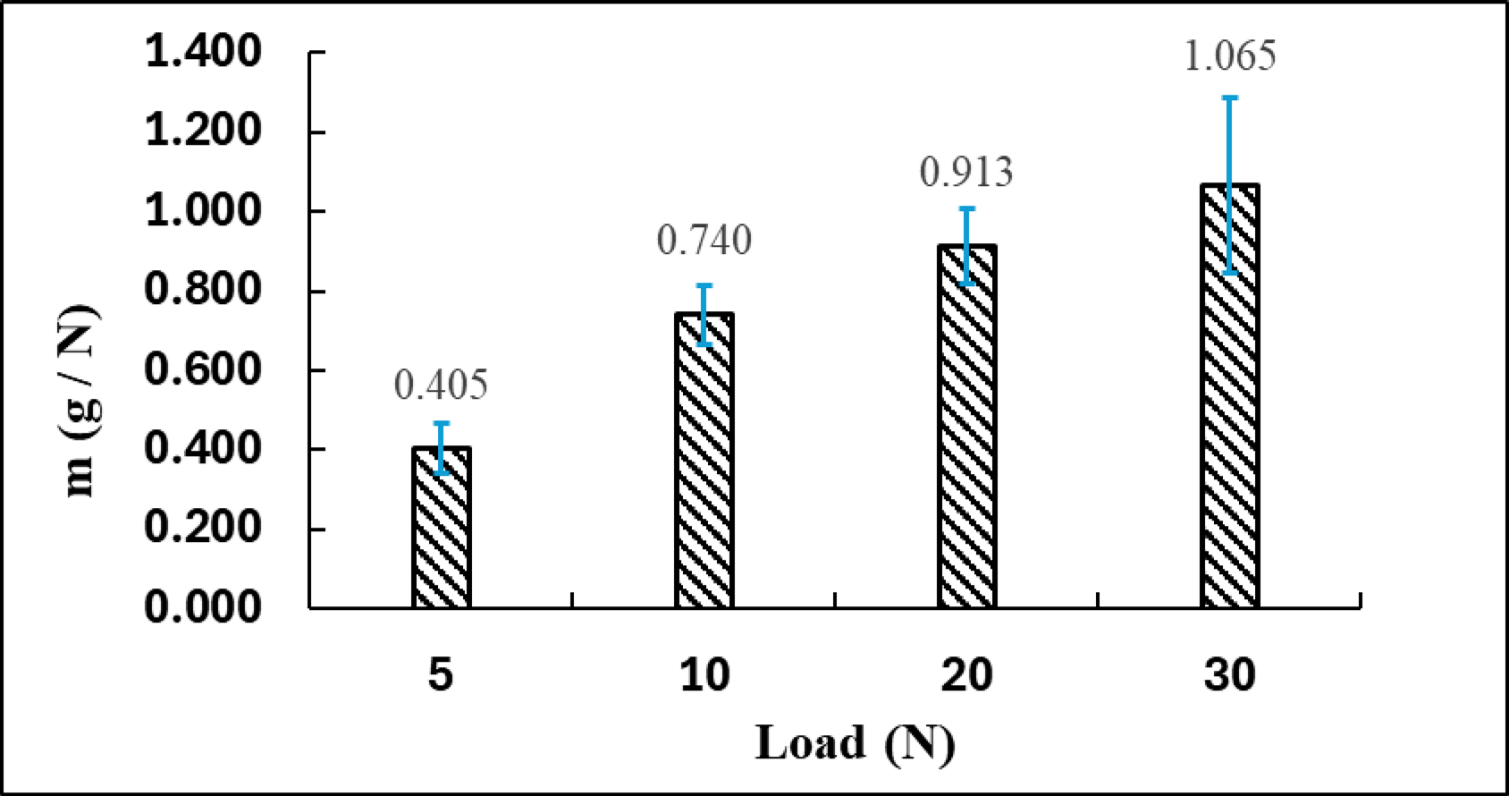
Influence of load on material wear performance.

Figure 8 illustrates the correlation between the applied normal load (N) and the specific wear rate of a brake pad composite, represented as m (g/N). The specific wear rate is a crucial parameter in tribological studies, quantifying material loss per unit load and serving as a key indicator for assessing the wear resistance of braking materials under different mechanical stress conditions^40^.

At a load of 5 N, the wear rate recorded is 0.405 g/N, representing the minimum value observed in the experiment. This indicates that at low contact pressures, the material experiences minimal degradation, probably because of limited heat generation and decreased mechanical disruption at the friction interface. The error bar at this load is minimal, suggesting reliable test outcomes.

With an increase in load to 10 N, the wear rate approximately doubles, reaching 0.740 g/N. This indicates an estimated increase of 82.7% relative to the wear observed at 5 N. The increase is due to elevated contact stresses, which cause more intense surface abrasion and the onset of microcracks, resulting in increased material removal. The increased wear rate at this stage may indicate the beginning of thermal softening or matrix degradation, which diminishes the composite’s resistance to additional wear^41^.

At a load of 20 N, the wear rate increases to 0.913 g/N, reflecting a 23.4% increase relative to the 10 N scenario. This trend indicates that material loss intensifies as load increases. The frictional heat and pressure may lead to partial degradation of the matrix or fiber-matrix interfaces, thereby diminishing the structural cohesion of the tribo-film formation. The increased wear rate exhibits a slight deceleration, indicating potential surface compaction or the development of a protective third-body layer that intermittently mitigates further wear.

At a maximum load of 30 N, the wear rate reaches 1.065 g/N, representing the highest value recorded in the study. This indicates a 16.7% increase relative to the 20 N result and a 162.7% increase in comparison to the wear observed at 5 N. The substantial error bar at this load signifies considerable variability in wear behavior, potentially resulting from uneven matrix degradation, localized thermal damage, or inconsistent formation of the third-body layer. The ongoing increase in wear rate with load indicates that the brake pad composite becomes more vulnerable to degradation under higher mechanical stress, notwithstanding potential enhancements in frictional performance, as evidenced by the COF figures.

Figure 8 demonstrates a direct correlation between applied load and wear rate, with specific wear increasing from 0.405 g/N at 5 N to 1.065 g/N at 30 N. The data suggests that although the composite may enhance frictional performance under elevated loads, this improvement is accompanied by greater material loss. The trade-off between friction and wear is a crucial factor in brake pad design. Optimizing material composition, including the balance of reinforcement content and matrix stability, is vital for ensuring long-term performance and durability in high-stress braking applications^41^.

The relationship between friction and wear at varying loads demonstrates a clear trade-off between performance and durability. Although the coefficient of friction increases from 0.697 at 5 N to 0.795 at 30 N, indicating stronger interfacial adhesion and asperity interlocking, this improvement is accompanied by a sharp rise in the specific wear rate from 0.405 g/N to 1.065 g/N. The enhanced frictional grip results from compaction and stabilization of a transient tribo-film; however, this same mechanism accelerates matrix degradation and micro-crack formation. When the applied load of 30 N was maintained for an extended duration, the friction coefficient initially remained high but gradually decreased to approximately 0.68 after 40 min, confirming that the tribo-film loses stability under prolonged mechanical and thermal stress. SEM observations at high load conditions Fig. 6-c reveal the onset of surface fatigue, fiber pull-outs, and microcracks, which are precursors to delamination and frictional fade. These findings confirm that while higher loads improve initial braking efficiency, they simultaneously accelerate wear and reduce long-term stability, establishing a clear friction–wear compromise in the composite material.

### Abrasive wear based on sliding velocity

**Fig. 9:**
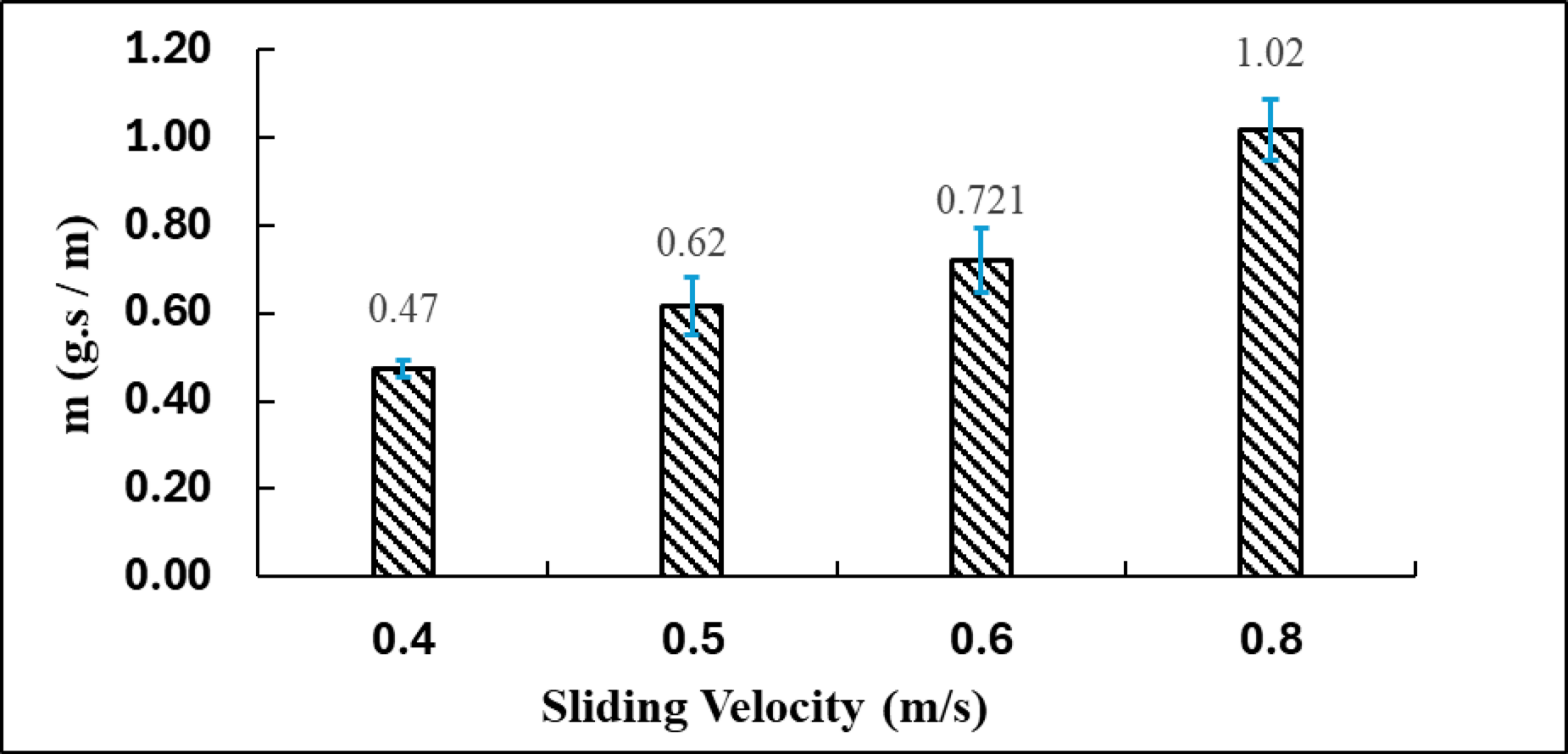
Relationship between sliding velocity and specific mass loss.

Figure 9 presents the relationship between sliding velocity (m/s) and the specific wear rate of the brake pad composite, expressed in g·s/m. As the sliding velocity increases from 0.4 to 0.8 m/s, the corresponding wear rate values plotted on the y-axis provide a quantitative assessment of material removal under progressively higher frictional energy input. The inclusion of error bars highlights the experimental variability associated with each measurement, reflecting the sensitivity of the wear response to dynamic changes in interface temperature and contact conditions. This visual representation enables a clearer interpretation of how increasing velocity influences the onset and severity of wear mechanisms within the composite material.

At a velocity of 0.4 m/s, the specific wear rate is 0.47 g·s/m, representing the minimum value observed in this dataset. This indicates that at reduced sliding speeds, the brake pad surface experiences comparatively mild abrasion. Reduced frictional heat and diminished mechanical interaction between contact surfaces likely lead to a decrease in material detachment. This value demonstrates a minimal error bar, reflecting significant consistency and stability in the measurements at this velocity^1^.

With an increase in sliding velocity to 0.5 m/s, the wear rate escalates to 0.62 g·s/m, reflecting an approximate increase of 31.9% relative to the wear rate at 0.4 m/s. The increase is due to enhanced surface interactions and the initiation of more severe micro-abrasive and fatigue wear. The rise in velocity results in enhanced frictional energy conversion into heat, which induces localized softening of the matrix and may expedite the removal of worn particles.

At a velocity of 0.6 m/s, the specific wear rate increases, attaining a value of 0.721 g·s/m. This indicates a 16.3% increase relative to the value at 0.5 m/s, implying a steady advancement in wear severity. The moderate error bar indicates stable yet somewhat variable test conditions, likely resulting from dynamic changes at the friction interface, including the formation or disruption of tribological films.

The maximum wear rate occurs at 0.8 m/s, recorded at 1.02 g·s/m, representing a 116% increase compared to the wear rate at 0.4 m/s. The notable increase indicates that high-speed sliding markedly enhances material loss. At this velocity, significant frictional heating occurs, potentially resulting in thermal degradation of the matrix and the failure of fiber–matrix bonding. The potential for thermal softening and oxidation increases, leading to the formation of unstable third-body layers and the generation of loose debris, which exacerbates wear^1^.

### Effect of different RPM on coefficient of friction

**Fig. 10:**
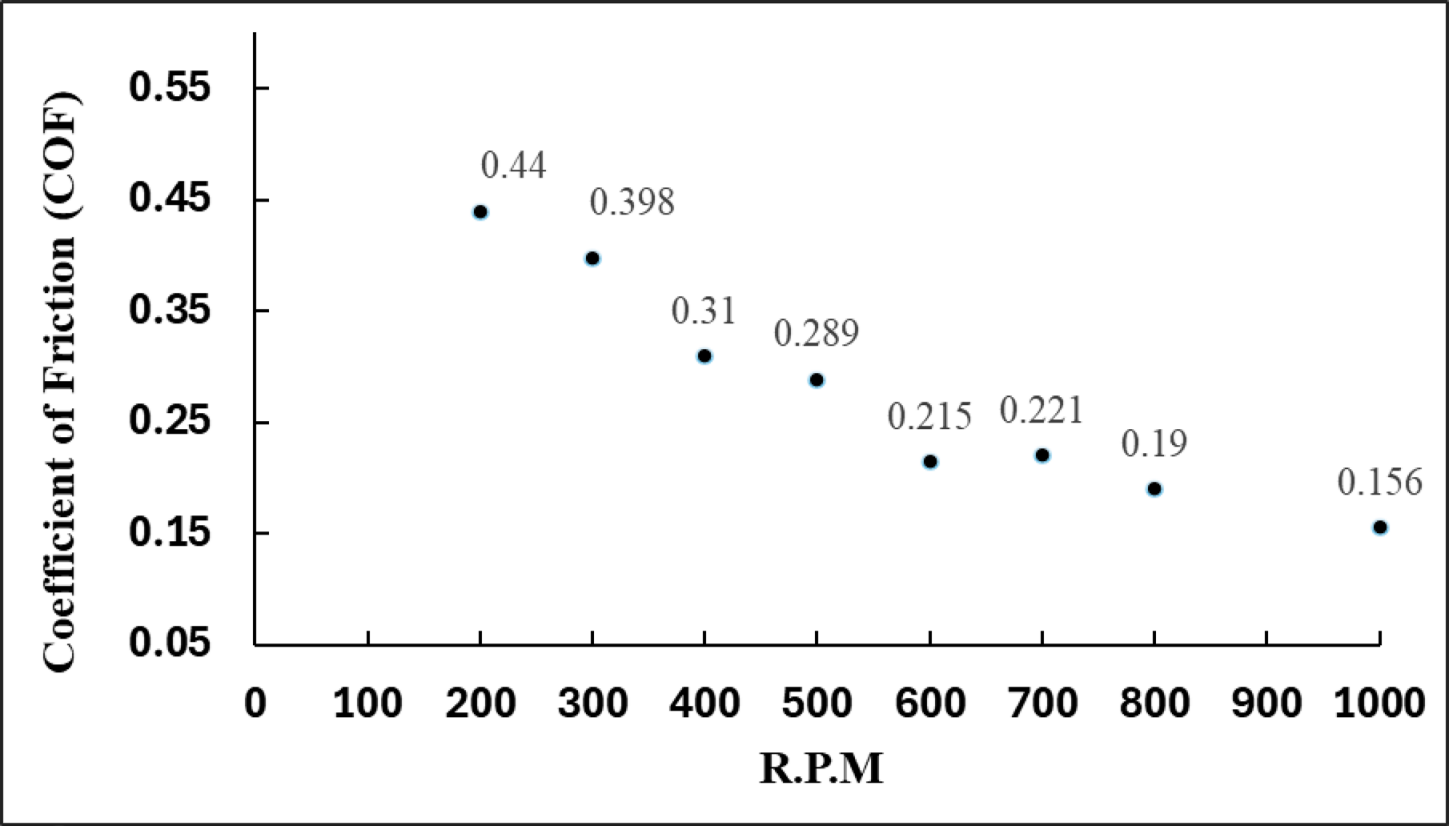
Frictional behavior of material at different RPM at 20 N load.

Figure 10 demonstrates the relationship between rotational speed (RPM) and the (COF) for a brake pad composite. At a low speed of 100 RPM, the (COF) is 0.44, representing the maximum value recorded in the dataset. This indicates that at low-speed operation, the composite surface retains effective interfacial grip, probably owing to reduced heat generation and minimal surface softening or degradation.

At an RPM of 200, the (COF) decreases marginally to 0.398, indicating a reduction of 9.5%. This suggests the onset of a declining trend, potentially attributable to initial thermal effects and alterations in the wear surface morphology, which begin to diminish the adhesion between the mating surfaces^33^.

As the speed increases to 400 RPM, the (COF) declines significantly, reaching a value of 0.31. This represents a 22.1% decrease from the initial coefficient of friction at 100 RPM, indicating that frictional heating intensifies, leading to the softening or weakening of the tribological film, which subsequently diminishes braking efficiency^33^.

At 500 RPM, the decrease to 0.289, maintaining the observed downward trend. This trend is particularly evident at 600 RPM, where the (COF) decreases to 0.215, reflecting a 51.1% reduction from the initial COF measured at 100 RPM. The significant decrease may result from a failure in surface integrity, leading to reduced surface resistance and diminished contact efficacy between the pad and the rotating surface.

At 700 RPM, the (COF) increases slightly to 0.221, indicating a minor rebound effect. This may result from a temporary reformation of a tribolayer or a more stable compaction of wear particles at this speed, which temporarily enhances surface interaction. This increase is marginal and not sustained.

At 800 RPM, the (COF) decreases to 0.19, and at the maximum tested speed of 1000 RPM, the COF attains its lowest value of 0.156, reflecting a 64.5% reduction from the value observed at 100 RPM. At elevated speeds, the surface is likely to undergo considerable thermal degradation, potential smearing of matrix material, and a reduction in mechanical interlocking, all of which contribute to decreased friction.

A significant change in wear regimes is seen by the correlation between rotational speed and COF. Wear is minimal (< 0.85 mg/N) and the COF is constant (0.63–0.72) at low speeds (200–400 rpm), suggesting good adhesion and the development of a protective third-body layer. Light abrasive wear is characterized by shallow grooves and modest particle pull-out in the SEM images taken at 200 rpm.

The surface morphology at 400 rpm shows a dense, continuous tribo-film made up of spread resin and consolidated wear debris. This film aids in frictional stability and bridges surface asperities. This is referred to in the literature as the “steady-state tribo-film regime,” in which the rate of compaction and debris production is equal.

At 800–1000 rpm, there is a noticeable decline. At first, the COF rises to about 0.80, but the wear rate surpasses 1.05 mg/N, signifying the beginning of severe fatigue, adhesive wear, and thermally driven softening. SEM images verify embedded debris, fractured areas, and melting-like smearing all indicators of tribo-film degradation. The heat deterioration of the resin and the fibers’ incapacity to provide interfacial support under fast cyclic loading are the main causes of this unstable friction state.

In the pin-on-disc tests, the applied rotational speed (200–1000 rpm) corresponds to sliding velocities ranging from approximately 0.25 to 1.26 m/s, depending on the wear track radius. At lower speeds (200–400 rpm), the brake pad surface exhibited stable frictional performance, with a coefficient of friction (COF) between 0.63 and 0.72 and low wear rates below 0.85 mg/N, attributed to the formation of a compact transfer film that reduced direct asperity contact. As the speed increased to 800–1000 rpm, the COF increase to 0.79–0.81, while the wear rate exceeded 1.05 mg/N, indicating thermal softening and matrix degradation leading to fiber pull-out and abrasive wear. This trend aligns with tribological principles observed in semi-metallic and composite friction materials, where higher sliding velocities increase interface temperature, weakening the resin matrix and promoting particle detachment.

Therefore, the observed relationship between speed and wear is not linear but thermally driven beyond a threshold velocity, excessive frictional heat accelerates surface fatigue and debris formation, adversely affecting braking efficiency. These findings highlight the critical role of rotational speed (and corresponding sliding velocity) in influencing the balance between friction stability and wear resistance. In future work, full-scale brake dynamometer tests will be conducted to validate these laboratory-scale results under real braking conditions.”

### Worn surface morphology of brake pad materials at varying rotational speeds

The SEM micrographs presented in Fig. 6 offer a comparative analysis of the worn surface morphology of a brake pad composite at different rotational speeds: (a) 200 RPM, (b) 400 RPM, and (c) 800 RPM. All images are captured at 1000× magnification, featuring a scale bar of 100 μm, which facilitates a detailed examination of the tribological behavior under varying dynamic conditions. The observations indicate distinct differences in wear mechanisms and surface evolution with increasing RPM^42^.

Figure 6a illustrates the worn surface at 200 RPM, characterized by a rough and heterogeneous morphology. The presence of sharp-edged reinforcement particles, some of which are partially extracted, in conjunction with grooves and micro-cracks, indicates that abrasive wear predominates at this low speed. The irregular distribution of wear debris and the absence of a continuous tribo-film suggest restricted thermal activation and mechanical energy input. The wear debris exhibits loose adhesion, and the lack of smeared areas indicates limited plastic deformation. The microstructure largely preserves its original characteristics, indicating mild wear accompanied by localized material detachment, likely caused by contact fatigue and mechanical interlocking among asperities^42^.

Conversely, Fig. 6b depicts the worn surface at 400 RPM, showing a markedly smoother and more compacted appearance. The wear track exhibits reduced particle pull-out and a lower incidence of cracks, along with the development of a more uniform and densified tribo-layer. This morphological transition indicates a change from abrasive to oxidative and adhesive wear mechanisms, facilitated by heightened frictional heat generation at this intermediate speed. The matrix phase seems to have initiated softening, which facilitates the smearing and bonding of fine wear debris into a semi-continuous protective layer. A tribological film protects the surface from additional abrasive wear and enhances the stability of the frictional interface. The wear is reduced in severity but increased in uniformity, indicating enhanced energy dissipation and a more stable sliding regime.

At 800 RPM, the worn surface (Fig. 6c) shows a dramatic transition into a severe thermo-mechanical degradation regime. The surface is dominated by extensive smearing, plastic flow, and large-scale morphological distortion, indicating that the matrix has undergone substantial thermal softening. Wear debris is compacted into thick, unstable agglomerates, and the darker regions suggest localized melting or resin pyrolysis caused by the intense frictional heat at this speed. Reinforcement particles become difficult to distinguish, as many are engulfed within the plastically deformed matrix, reflecting a collapse of the microstructural framework. These features collectively signify a shift toward severe adhesive wear, thermo-oxidative degradation, and fatigue-driven surface tearing. The tribo-film at this stage is highly unstable, prone to cracking and delamination, which accelerates material removal. Consequently, the 800 RPM condition exhibits the most destructive wear behavior, marked by pronounced structural breakdown, loss of surface integrity, and a substantial reduction in the composite’s ability to maintain a stable frictional interface.

The transition from 200 to 800 RPM indicates a shift in wear behavior from mechanical abrasion to thermo-mechanical deformation and fatigue. At 200 RPM, wear is minimal and localized; at 400 RPM, the surface shows the formation of a protective tribo-film; and at 800 RPM, the surface experiences significant damage from excessive thermal and mechanical stress^43^. The findings underscore the significance of choosing operational speeds that enhance both friction and wear resistance in braking systems.

The worn surface morphology at different rotational speeds is shown in Fig. 6a–c. At 200 RPM, the surface exhibits scattered grooves and partially detached particles, indicating mild abrasive wear. At 400 RPM, a smoother and more compact region is observed, representing the formation of a continuous tribo-film that bridges adjacent asperities and partially covers the exposed reinforcement particles. This compacted layer consists of fine oxidized debris and resin smearing, suggesting that localized frictional heat promotes compaction and adhesion of wear particles into a protective film. At 800 RPM, the tribo-film becomes unstable and discontinuous due to excessive thermal and mechanical stress, leading to surface softening and large-scale delamination. The contrast between the dense tribo-layer at 400 RPM and the degraded matrix at 800 RPM supports the proposed transition from a protective to a destructive wear regime at higher speeds^9^.

After tribological testing, surface densification and increased hardness (HRC) confirm that repeated sliding induces work-hardening and tribo-layer compaction. The synergy between SiC (abrasive), MgO (thermal stabilizer), and 3-mm aramid fibers (reinforcement) supports this adaptive behaviour. However, at elevated speeds, this synergistic effect is compromised due to extensive thermal damage, explaining the sharp rise in wear observed at 800–1000 rpm.

## Conclusions

- The developed fiber-reinforced brake pad composite demonstrated a stable coefficient of friction (COF) across moderate operating loads and speeds, with optimal frictional performance achieved between 10 and 20 N and 200–400 rpm.
- Increasing the applied load from 5 N to 30 N enhanced the COF by approximately 14%, due to improved asperity interlocking and tribo-film compaction. However, this improvement was accompanied by a 162% increase in wear rate, highlighting a friction–wear trade-off.
- The sliding velocity significantly influenced tribological response. The COF rose by 20.2% (from 0.663 to 0.797) as velocity increased from 0.4 m/s to 0.8 m/s, while the wear rate more than doubled, indicating that excessive thermal input leads to matrix degradation.
- Higher rotational speeds (above 800 rpm) caused a steady decline in COF and severe surface damage due to thermal softening and unstable tribo-film formation.
- SEM observations confirmed a transition in wear mechanisms:
- At 200 rpm → localized abrasive wear.
- At 400 rpm → formation of a dense protective tribo-film.
- At 800 rpm → breakdown of the film with pronounced adhesive and fatigue wear.
- Hardness results showed increased HRC values after testing, attributed to friction-induced surface densification and work-hardening. Reinforcements of SiC, MgO, and 3-mm aramid fibers collectively improved thermal stability, stress distribution, and wear resistance.
- Overall, the composite exhibited strong frictional stability under moderate operating conditions but optimizing the ratio of reinforcing fillers and enhancing thermal conductivity are recommended to minimize wear at higher loads and speeds.

## Data Availability

The datasets used and/or analysed during the current study available from the corresponding author on reasonable request.

## Abbreviations

COF: Coefficient of Friction, Dimensionless
RPM: Rotational Speed, revolutions per minute (rpm)
HRC: Rockwell Hardness (C-scale), Hardness unit
N: Normal Load, Newton (N)
m: Specific Wear Rate, g/N or g·s/m
SiC: Silicon Carbide (reinforcing phase)
MgO: Magnesium Oxide (reinforcing filler)
SEM: Scanning Electron Microscope, Imaging method
FESEM: Field Emission Scanning Electron Microscope, High-resolution imaging
LSCM: Laser Scanning Confocal Microscopy, Surface profiling technique
SAE J661: Standard testing procedure for brake materials, Society of Automotive Engineers
g·s/m: Specific wear rate per sliding distance and time, grams per second per meter
